# Diagnostic Dental Radiation Risk during Pregnancy: Awareness among General Dentists in Tabriz

**DOI:** 10.5681/joddd.2011.014

**Published:** 2011-06-14

**Authors:** Tahmineh Razi, Leila Bazvand, Morteza Ghojazadeh

**Affiliations:** ^1^ Assistant Professor, Department of Oral and Maxillofacial Radiology, Faculty of Dentistry, Tabriz University of Medical Sciences, Tabriz, Iran; ^2^ DDS, Private Practice, Tabriz, Iran; ^3^ Assistant Professor, Department of Physiology, Faculty of Medicine, Tabriz University of Medical Sciences, Tabriz, Iran

**Keywords:** Pregnancy, protection, radiation

## Abstract

**Background and aims:**

Pregnant women often do not receive proper dental care in emergency visits due to a lack of awareness of the effect of radiation doses and the involved risks for the fetus. The aim of the present study was to assess the awareness of general dentists practicing in Tabriz, Iran, of the risks involved during exposure to diagnostic dental radiation in pregnant women.

**Materials and methods:**

In this descriptive/cross-sectional study, 250 general dentists, who had attended continuing education courses under the supervision of the Faculty of Dentistry, filled out questionnaires on their awareness of radiation risks. Data was analyzed by Spearman's correlation coefficient test.

**Results:**

The mean of correct answers was 6.47±1.66, with the least and highest correct answers of 2 and 10, respectively. The highest and the lowest levels of awareness were related to the use of a lead apron (92%) and a long rectangular collimator (3.2%), respectively. There was a statistically significant correlation between the age of practitioners and awareness of radiation risks (P=0.02). However, no statistically significant correlation was observed between job experience (P=0.25) and the number of continuing education courses attended (P=0.16) and awareness of radiation risks.

**Conclusion:**

The studied population of dentists does not seem to have the sufficient knowledge regarding the diagnostic dental radiation risk during pregnancy. Further educational courses and pamphlets are recommended for increasing their awareness of this subject.

## Introduction

Pregnant women often pay visits to dental offices for pain and infections associated with their teeth. Such patients need dental treatment and in most cases the radiograph of the involved tooth is required. It is repeatedly reported that dentists postpone dental treatments to the period after delivery because they do not have sufficient knowledge of the low doses involved in diagnostic dental radiation. The delay in treatment might have adverse effects on the mother and the fetus.

Most of the biologic responses to radiation occur during the first two weeks of pregnancy, which is a period when the mother is unaware of her pregnancy, and these responses lead to miscarriage of the fetus. Therefore, there is no concern about congenital abnormalities during the first two weeks of pregnancy. Spontaneous abortion subsequent to radiation during the first two weeks of pregnancy at doses less than 25 rads (250 mGy) is improbable.^1^

The results of several studies suggest that if there is a response to diagnostic radiation during late second semester, the main problem will be a malignant disease during childhood.^1^ Such a response to radiation during pregnancy is the result of a very high dose. Approximately 5% of live births show a congenital abnormality without any exposures involved. It is estimated that there is a one-percent increase in congenital abnormalities subsequent to an exposure of 10 rads (100 mGy) of fetal dose. Since diagnostic doses are less than 10 rads in dentistry, such abnormalities cannot be attributed to dental diagnostic doses.^2,3^

Kusama et al^4^ indicated that the fetus does not directly receive radiation doses during head and chest diagnostic exposures and that the absorbed dose was estimated at less than 0.01 mGy. It was concluded that in women who were unaware of their pregnancy and who had been exposed to radiation, there is no need for pregnancy termination when the exposure dose to the fetus is less than 100 mGy.^4^

Nonetheless, no radiography procedure should be carried out on pregnant women unless there is an absolute necessity. All techniques for minimizing the absorbed dose should be undertaken when such radiographs are mandated. Radiographs should be provided with well-collimated beams in precisely-protected shields. A high-kVp technique is appropriate in such cases.^1^ Pregnant women can receive dental treatment considering the above-mentioned principles and the possibility of carrying out radiographic techniques during pregnancy by observing protective measures, awareness of the safest period for radiography, and doses involved in diagnostic procedures. Use of lead aprons can reduce radiation doses to gonads up to 98%. In a pregnant woman maternal tissues covering the fetus reduce fetal dose to 30% of the abdominal skin dose.^1^

The aim of the present study was to evaluate awareness of radiation risks for pregnant women among general dentists in a large city in Iran. The results of the present study might help improve the knowledge of dentists regarding the effect of radiation in pregnant women and the possibility of carrying out some dental treatment procedures by observing principles of radiation protection.

## Materials and Methods

The subjects in this descriptive/cross-sectional study consisted of 250 general dentists practicing in Tabriz, Northwest Iran. All of the eligible subjects were included in the study without discrimination. Data were collected by distributing questionnaires among the subjects who had taken part in continuing education courses. The questionnaire had been designed by the researcher, which consisted of 15 questions about the awareness of the risk of dental radiation in pregnant women. The questions included participants’ age, years in practice, taking part in continuing education courses and the data regarding their knowledge of the safe dose for the fetus, radiation protection principles, the film type and the radiographic techniques, all of which result in reduced radiation dose to pregnant women. The validity of the questionnaire was evaluated by content validity method and its reliability was approved by Cronbach’s Alpha coefficient of 0.78.

Anonymity and confidentiality of the answers were emphasized when handing out the questionnaires.

Descriptive statistical methods (mean ± standard deviation), frequency (percent) were applied to date. Spearman’s correlation coefficient test was also employed. SPSS 13.0 computer software was used for the analyses. Statistical significance was defined at P<0.05.

## Results

The mean age of the subjects was 37.78 ± 6.08 years (range: 25–54 years). The mean job experience was 10.65 ± 6.98 years (range: 5–30 years). The mean frequency of participation in continuing education courses was 10.28 ± 6.09 times (range: 0–20 times) during their profession.

**Figure 1:**
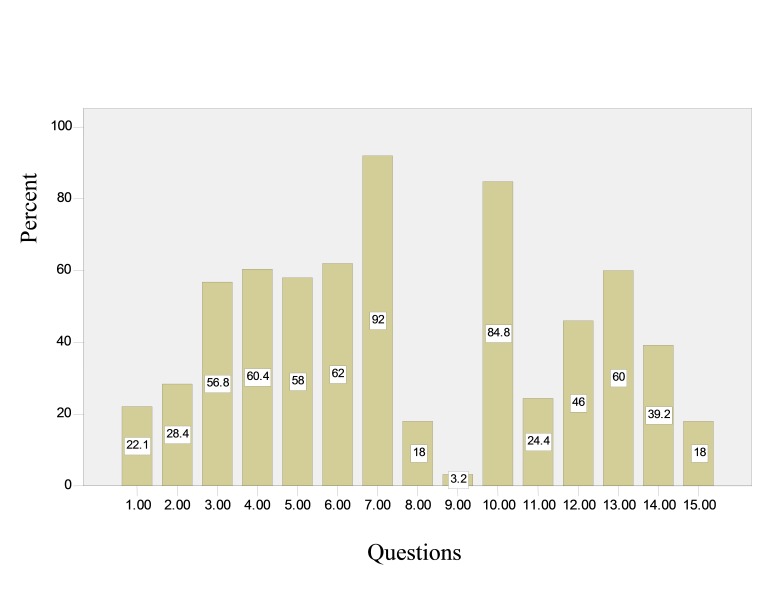
The percent of correct answers to the questions

Mean number of correct answers by the subjects regarding awareness about radiation was 6.47 ± 1.66. The least and highest correct answers were 2 and 10, respectively. Figure 1 show the percent of dentists’ correct answers to the 15 questions included.

The results showed that there was an inverse correlation between dentists’ age and their level of awareness (rho= −0.34, n=250, P=0.02).

In addition, no rho correlation was observed between job experience (P=0.25) and the times of participations in continuing education courses on one hand (P=0.16) and the number of correct answers on the other hand.

## Discussion

The results of the present study revealed that only 22.1% of dentists were aware that no chromosomal abnormalities occur subsequent to exposure to diagnostic radiation doses. Chromosomal abnormalities are proportional to the radiation doses absorbed.^2^ There is no data regarding the genetic effects of radiation in human beings. In 1927, the Nobel prize winning geneticist J. Muller reported the effect of radiation on Drosophila, indicating that radiation does not change mutation quality but increases the rate of spontaneous mutations.^1^ Of the dentists under study only 28.4% (71 subjects) were aware that diagnostic radiation doses do not result in developmental anomalies or mental retardation in fetuses; there are no reports of developmental anomalies or mental retardation in fetuses exposed to diagnostic radiation doses.^5^ Around 56.8% (142 subjects) were aware of the period in which the fetus is the most sensitive to radiation. All the observations indicate that the first semester is the most sensitive period during pregnancy,^1^ and exposure threshold for the development of definitive defects increases after main organogenesis period.^6^

A total of 60.4% (151 subjects) of the participants believed that diagnostic radiation doses result in neoplasms in the later stages of life. Data regarding radiation-dependent neoplasms has been derived from populations exposed to high-dose radiations; however, low-does radiation can initiate the neoplasmic process in individual cells.^1^ Regardless of the effect of heredity, individual exposure to diagnostic radiation of intra-oral techniques increases the incidence of new fatal neoplasms by only 0.2%.^2^

However, the results of a study carried out by Brent^7^ have demonstrated that high radiation doses increase neoplasm risk; there is a possibility that increased carcinogenic risk is of no concern in diagnostic radiology.

A total of 58% (145 subjects) of the dentists under study were aware of the threshold radiation doses (25 rads) for pregnancy termination. Radiations threshold for the development of congenital defects during the most sensitive period is 0.2 Gy and the threshold for growth retardation and abortion is much higher.^6^ Response to radiation during pregnancy requires a very high dose. No response will be elicited at doses less than 25 rads (250 mGy).^1^

Almost 62% of the subjects were aware of the necessity of questioning the subject about pregnancy before radiographic procedures. No pregnant women should undergo radiographic procedures unless there is an absolute necessity for it. When such procedures are undertaken, all the precautions should be exercised to minimize the radiation dose.^2^ A total of 92% of the subjects were aware of the necessity of the use of lead aprons during radiography. The use of a lead apron reduces the absorbed dose of most organs and tissues to almost zero.^1^ In addition, the use of a lead apron reduces radiation dose to gonads up to 98%.^2^ A total of 18% of the subjects believed it is necessary to use F-speed films. High-speed films are efficient in reducing radiation doses.^2^ Recent research indicates that F-speed films have a similar or higher contrast compared to Ekta-speed Plus films and reduce patient exposure up to 20%.^8,9^

Only 3.2% (8 subjects) of the participants were aware of the use of long rectangular collimators. Size and shape of x-ray beams are important factors involved in the radiation dose absorbed by the patient.,^11^ In periapical radiographic technique, horizontal limiting of radiation field to accommodate film size (rectangular collimator) is suggested.^12^ Some studies have reported that no dental practitioner makes use of a rectangular collimator;^13,14^ however, some other studies have reported that some dentists make use of such collimators.,^16^ A rectangular collimator with a 3.5 × 4.4 cm^2^ outlet reduces exposure area and patient skin dose up to 60% compared to a round collimator.^2^

A total of 84.8% (212 subjects) of the dentists under study were aware of the quality of developing solutions and the role of their timely renewal in the patient exposure. If films are not properly processed, patient exposure will increase. A total of 30% of repetitions of radiographic techniques is due improper film density, which is directly related to conditions during film processing.^2^

The results of the present study indicated that there was an inverse relationship between aging and awareness of diagnostic radiation risks in pregnant women among the studied population. The number of years in practice and the number of times a dentist has took continuing education courses did not significantly affect dentists’ awareness.

Since the awareness of dentists has a direct effect on their behavior, re-training courses should be planned to increase their knowledge of the safety of radiographic procedures and the use of protective techniques in pregnant women.

## Conclusion

According to the results, it can be concluded that general dentists have insufficient knowledge of the safety of diagnostic radiation doses in pregnant women and only about a one-fourth (28.4%) of them were aware that diagnostic doses result in no developmental or mental problems in the fetus. Dentists had an acceptable level of awareness regarding principles of radiation protection. They were also aware of the role of developing solutions and their timely renewal in reducing patient exposure.
